# 3D imaging of human organs with micrometer resolution - applied to the endocrine pancreas

**DOI:** 10.1038/s42003-021-02589-x

**Published:** 2021-09-10

**Authors:** Max Hahn, Christoffer Nord, Maria Eriksson, Federico Morini, Tomas Alanentalo, Olle Korsgren, Ulf Ahlgren

**Affiliations:** 1 Umeå Centre for Molecular Medicine, Umeå University, Umeå, Sweden; 2 Department of Immunology, Genetics and Pathology, Uppsala University, Uppsala, Sweden

**Keywords:** Diabetes, Optical imaging, 3-D reconstruction, Islets of Langerhans

## Abstract

The possibility to quantitatively study specific molecular/cellular features of complete human organs with preserved spatial 3D context would have widespread implications for pre-clinical and clinical medicine. Whereas optical 3D imaging approaches have experienced a formidable revolution, they have remained limited due to current incapacities in obtaining specific labelling within large tissue volumes. We present a simple approach enabling reconstruction of antibody labeled cells within entire human organs with preserved organ context. We demonstrate the utility of the approach by providing volumetric data and 3D distribution of hundreds of thousands of islets of Langerhans within the human pancreas. By assessments of pancreata from non-diabetic and type 2 diabetic individuals, we display previously unrecognized features of the human islet mass distribution and pathology. As such, this method may contribute not only in unraveling new information of the pancreatic anatomy/pathophysiology, but it may be translated to essentially any antibody marker or organ system.

## Abstract (web summary)

Hahn et al. present a method to visualize the endocrine human pancreas in 3D and calculate volumetric data. Using immunolabeling to visualize targets of interest and in reconstructing large tissue parts from imaged cm3-sized tissue blocks, they use their method to reveal previously unknown morphological differences in the endocrine pancreas affected with type 2 diabetes.

## Introduction

A range of mesoscopic imaging approaches are currently available for optical deep tissue imaging of biological samples, including optical projection tomography (OPT)^1^ and light-sheet fluorescence microscopy (LSFM)^2–4^ (for comparative review see Liu et al.^5^). These technologies can provide micrometer resolution 3D renderings of optically cleared tissues on the mm-cm scale. Whereas a variety of efficient methodologies have been developed to render tissues optically transparent for this type of imaging^6–8^, a major limitation for their utility to provide a holistic view of cellular/molecular expression patterns in human organs is related to challenges in obtaining specific labelling that cannot be implemented in humans, including methods for transgenic expression of fluorescent proteins or other methods that have limited target selectivity. To fully exploit the potential of these approaches for studies of human pathophysiology, a method that could selectively label any target of choice would be highly desirable.

In this report, we provide a simple approach for human organ reconstruction that enables both 3D rendering and quantification of the investigated cell types. The technique can be performed by presently available mesoscopic imaging devices and builds on the stitching of antibody labelled cm^3^-sized tissue blocks back into 3D space. As an object of investigation, to demonstrate the utility of this approach, we turned to the human pancreas with its complex compound endocrine/exocrine organization. The endocrine cells, organized into the islets of Langerhans, constitute only a fraction (≈1–2%) of the pancreatic mass but are scattered in vast numbers throughout the exocrine parenchyma^9,10^, making assessments of their distribution, mass, and local pathology a major challenge when applying currently available techniques. Solving such quantitative issues would allow for regressive indications on the endocrine organization and the relation to β-cell function of the organ. In this proof of principle study, we analyzed pancreatic tissue encompassing over 200.000 islets and provide information about normal and aberrant islet composition, including the localization of regions with high islet density and of islets with internal hemorrhaging.

## Results

**Fig. 1:**
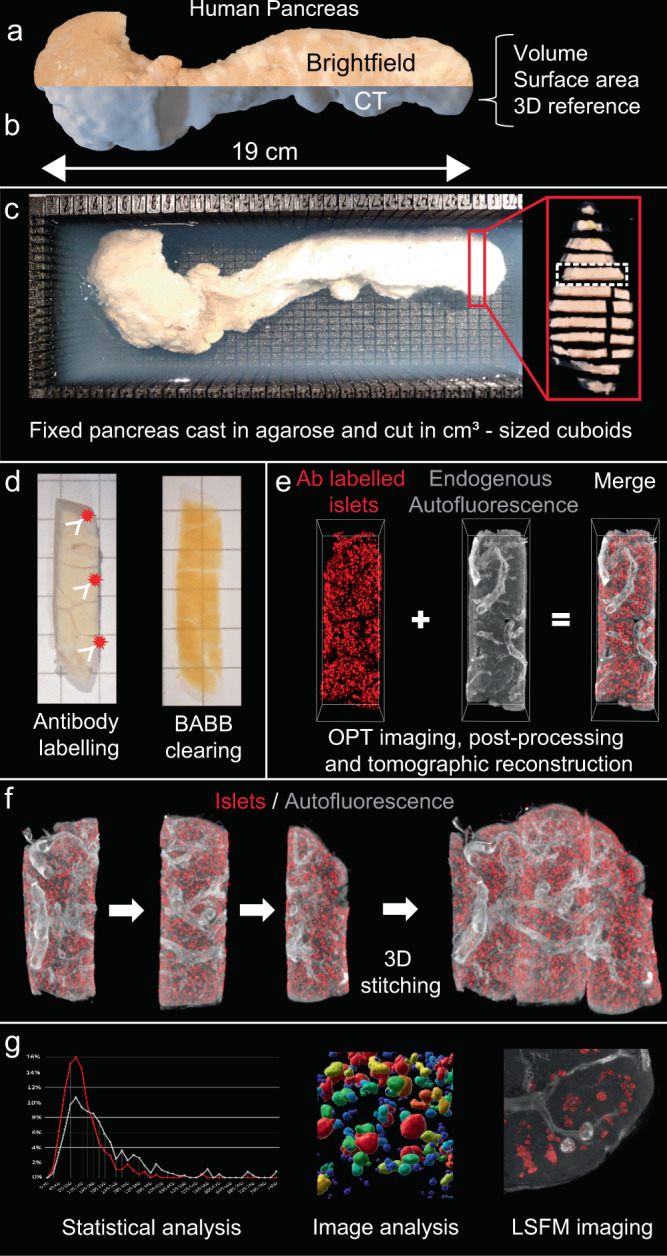
A pipeline for quantitative molecular 3D imaging of human organs. Schematic outline illustrating the process of tissue processing and image analysis as applied to the human pancreas. By embedding fixed intact, CT-scanned fixed pancreas (**a**, **b**) from diseased donors in agarose, cm3-sized tissue cuboids are sliced and extracted from a 3D printed matrix (**c**). The cuboids are subjected to whole-mount immunohistochemistry and scanned by Near-infrared - OPT^31^ (**d**, **e**). The resultant scan data is post-processed by schemes for image fusion-OPT^37^ and CLAHE^32^, and the segmented tomographic data is stitched back into 3D space (**f**) (see also Fig. 2). Hereby, statistical and computational image analyses of the islet mass distribution can be performed including information of the islet’s individual 3D coordinates, volumes, and shapes within the context of the entire gland (**g**). In addition, AF features of the tissue may be used to generate contrast for vessels and other structures (see Fig. 3). Since the tissue processing protocols are fully compatible, complementary high-resolution analyses can be performed by LSFM imaging. In all, the produced data provide proof of principle for the applicability of the approach to reconstruct the amount, volume, and distribution of antibody labeled features throughout the volume of the organ.

We created a pipeline (see Fig. 1) wherein cm^3^-sized pancreatic tissue cuboids from diseased donors are isolated with maintained spatial context (Supplementary Fig. 1). By subjecting these to improved antibody labelling protocols (here against insulin) and mesoscopic imaging (here OPT and LSFM), individual islet volumes and their spatial 3D coordinates could be determined (see Supplementary Fig. 2 for image processing pipeline). When stitching the individual datasets together back into 3D space, we show how the method can be applied to generate complete 3D maps of immune labelled objects throughout the human pancreas while maintaining volumetric and spatial information of the individual islets. Additionally, we show that the auto-fluorescent tissue properties can be used to extract 3D information of organ pathology. Of note, although OPT was used as the primary technology in this study, the implemented tissue processing protocols are fully compatible with LSFM (see Supplementary Movie 1 and 2), applied here to obtain complementary high-resolution assessments. However, sole LSFM may be used for the same type of assessments, ideally when compensation for non-isotropic voxels is implemented^11^.

### Comparative assessments of islet 3D distribution

**Fig. 2:**
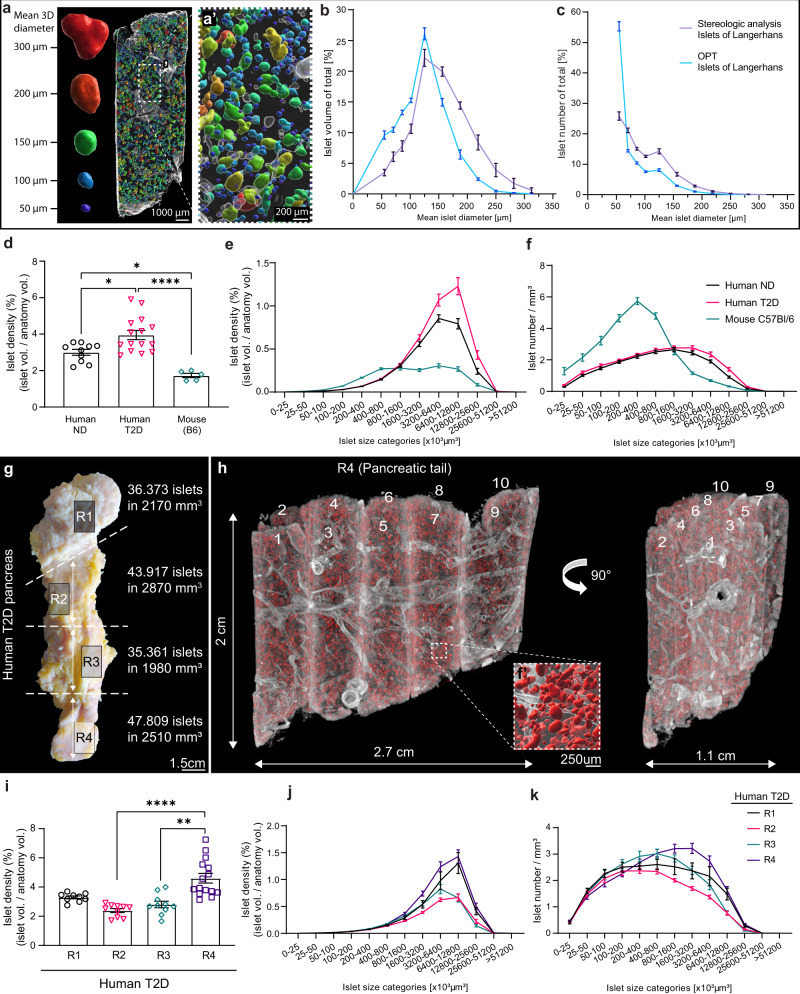
Quantitative, volumetric and 3D-spatial assessments of the islets of Langerhans distribution in the human pancreas. **a** Example of tissue cuboid in which the labeled islets have been colored according to size, displaying “absolute” volumetric data omitting the need for extrapolation of 2D data and inaccuracies in such estimates due to non-sphericity of the islets. **b**, **c** Islet size distributions from OPT data of 33.637 islets from a non-diabetic donor, re-calculated to mean islet 3D diameters, compared to data obtained by point counting morphometry (table 1 in Hellman 1961^12^), showing the islet volume per size category (**b**) and the islet number per size category (c) of the total. **d**–**f** Islet densities comparing tissue from a non-diabetic (ND) donor, a type 2 diabetic (T2D) donor, and from C57/Bl6 mice (8-weeks, *n* = 5) encompassing 33.637, 47.809, and 30.667 islets respectively, displayed as mean densities (**d**) and density per islet size category (**e**), and islet count per mm3 of tissue for each size category (**f**). Note, densities in (**e**) and (f) are normalized against the outline anatomy of the tissue (see methods). **g** Photomicrograph of a T2D donor pancreas illustrating the origin of the analyzed tissue (10–15 tissue cuboids per region R1-4), their islet count, and volume. **h** OPT image of 10 stitched tissue cuboids (from R4) labelled for insulin (red), stitched back into 3D space. The vessels and ducts (grey) have been reconstructed based on their AF properties. Inset shows high magnification view corresponding to box indicated by a broken line, in which the islets have been iso-surfaced for the perception of 3D depth. **i**–**k** Average islet volume density (**i**), average islet volume density per size category (**j**), and average islet count per mm^3^ of tissue for each size category (**k**) in regions R1-4 of the pancreas (displayed in (**g**)). Islet densities are normalized against the actual tissue volume, i.e., the volume of vessel lumens, etc. has been extracted (see methods). Significance was tested using a one-way ANOVA (**d**) and a non-parametric Kruskal–Wallis test (**i**). Error bars represent SEM. *P ≤ 0.05, **P ≤ 0.01, and ***P ≤ 0.0001, respectively.

Initially, we analyzed the islet size distribution of pancreatic tissue encompassing 33.637 islets from the pancreatic tail of a non-diabetic (ND) donor and re-calculated the individual islets 3D volumes to their average diameters to compare our data sets with stereological data (see methods). Assessments of the islets cross-sectional diameters showed no difference between OPT, LSFM, and histological tissue sections of the same specimens (Supplementary Fig. 3, *p* > 0.8, mean diameter difference −3.62% and 0.137% for OPT and LSFM versus sections respectively). Compared to earlier comprehensive point counting morphometric studies^12^, our data display striking similarities, both regarding the relative distribution of islet mass and islet number per size category (Fig. 2a–c, Supplementary Movie 3). However, compared to the stereological data, our OPT analysis was able to detect a higher number of islets in the smallest size category, accentuating the challenge of accurately representing the full extent of 3D features based on the extrapolation of 2D data. OPT data are based on actual 3D volumes within the investigated tissue. Therefore, plotting the islets’ distribution as volume size categories omits the requirement for sphericity/non-sphericity compensation. Next, we compared the ND islet volume distribution as measured by OPT, with the islet volume distribution from the corresponding tail part of a type 2 diabetic (T2D) donor and of entire pancreata of C57Bl/6 mice (*n* = 5), encompassing 33.637, 47.809, and 30.667 islets for ND, T2D, and B6 mice, respectively (Fig. 2d–f). Our data show that the islet density in the human pancreas investigated is significantly higher when compared to the mouse (*p* = 0.0145 for ND and *p* < 0.0001 for T2D). The latter has a relatively higher proportion of smaller islets. Noteworthy, there were relatively larger islets present in the T2D samples than in that of the ND samples. Furthermore, sphericity analyses on islets of different size categories, showed that large islets (>9 × 10^6^ μm^3^) from the T2D sample is significantly less spherical in shape (*p* < 0.01) compared to islets of the same size categories from the ND sample (see Supplementary Fig. 4), indicating that changes in islet morphology may be associated with T2D development in humans. Notwithstanding the limited sample size, these analyses demonstrate the applicability of our approach for comparative assessments of “absolute” islet volume distributions and islet morphology between pancreata with different disease history, as well as between species.

### Large scale 3D reconstruction of antibody labelled human pancreas

By expanding our analyses to include 9,53 cm3 of tissue from a T2D donor pancreas, encompassing in total 163.460 islets, we compared the islet volume distribution pattern in four different regions of the gland (Fig. 2g, region (R) 1–4). Based on OPT data of tissue cuboids stitched back into 3D space (Fig. 2h, Supplementary Movie 4, see methods), our data show that in the investigated pancreas, the islet volume density was significantly higher in R4 (tail) compared to R2 and 3 (Fig. 2i, *p* < 0.0001 and *p* = 0.0015 for R2 and 3 respectively). Furthermore, the total islet volume in R1 (head) and R4 (tail) was constituted by a relatively higher fraction of large islets compared to R2 and R3 (Fig. 2j, k). These analyses indicate that the islet distribution in the human pancreas, in similarity to its rodent counterpart^13^, is far from homogenous and is characterized by significant regional differences.

### Islet distribution in the human pancreas is highly heterogenous with high islet density areas in the organ periphery

**Fig. 3:**
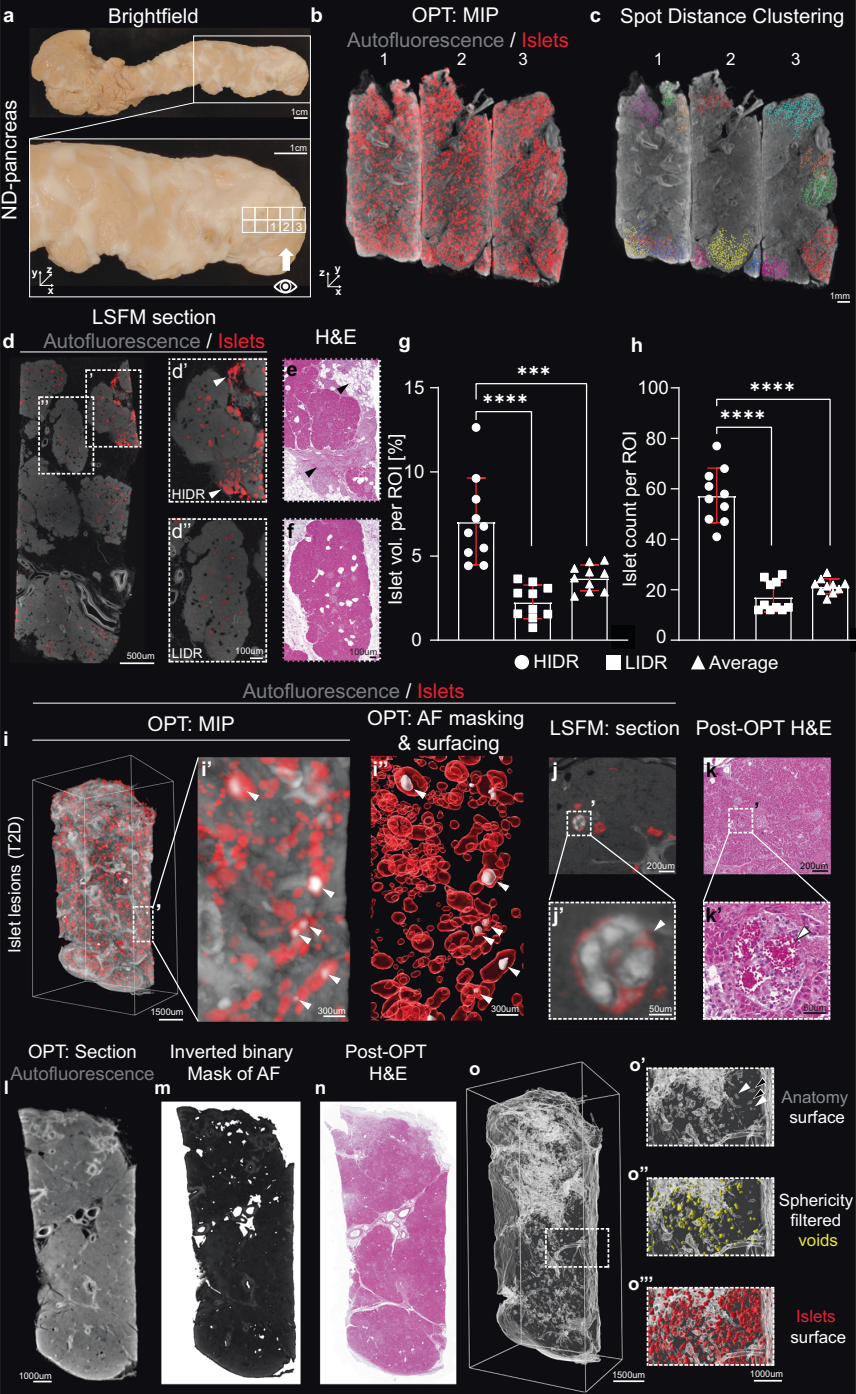
Assessments of islet clustering, islet hemorrhaging and non-fluorescent features of the human pancreas. **a** Photomicrographs of an intact human pancreas from an ND donor illustrating viewing angle in (**b**, **c**). See “eye” in the inset. **b** OPT maximum projection intensity (MIP) image of three tissue cuboids (from (**a**)) stained for insulin (red) and autofluorescence (AF, grey), stitched together in 3D space. **c** Representative spot analysis of islets in the tissue cuboids (seen in (**b**)), showing the appearance of HIDR´s (>100 islets per cluster) localized towards the organ surface. Within each cluster (pseudo colored), the islets are located within 300 μm from its nearest neighbor (see methods and text for details). **d** LSFM section showing a HIDR (d’, white arrowheads) and a LIDR, (d”). **e**, **f**, H & E staining of tissue sections corresponding to (d´ and d´´), obtained post-OPT and LSFM imaging. Note the appearance of HIDR’s in fibrotic/adipocyte-rich areas (see black arrowheads in **e**). **g**, **h**, Quantitative assessment of average islet volume densities (**g**) and islet counts (**h**) per ROI volume in HIDRs and LIDRs (*n* = 10 tissue cuboids) corresponding to white boxes in (**a**). **i** OPT-based MIP 3D rendering of internal islet hemorrhaging based on autofluorescence from red blood cells (RBC´s). Islets (red) are visualized based on insulin staining whereas the signal from RBCs provides a sufficient signal-to-noise ratio to enable segmentation of hemorrhaging (white, arrowheads in i´ and i´´). **j** Section from an LSFM scan section displaying RBC AF in an islet with hemorrhaging. **k** H&E staining of a section corresponding to (**j**) obtained post 3D imaging showing the correlation between the AF signal of RBCs and internal islet RBCs. **l**, **m** Tomographic section (**i**) from an OPT scanned tissue cuboid and its corresponding image when subjected to a binary mask and inverted (**m**) for visualization of hypointense regions. **n**, H&E stained section obtained post 3D imaging corresponding to (**l**, **m**). **o**, Anatomical iso-surface segmentation of the hypointense regions from the sample in (**l**–**n**). Insets (o´-o´´´) shows high magnification corresponding to the volume indicated by a broken white line in (**n**) and shows the hypointense volumes without (o´) and with filtering for spherical objects (yellow, o´´), whereas (n´´´) shows a 3D rendering of the islets based on insulin staining for reference. Error bars represent SEM. *** and **** represent P ≤ 0.001 and ≤ 0.0001, respectively. Abbreviations; AF autofluorescence, H&E Hematoxylin, and eosin, HIDR high islet density region, LIDR low islet density region, LSFM light-sheet fluorescence microscopy, OPT optical projection tomography, ROI region of interest.

The possibility to obtain full tomographic data sets, including shape, volume, and 3D coordinates for each individual islet, enables statistical assessments and image analyses that are extremely challenging, or even impossible, with stereological techniques. As depicted in Fig. 3, our technique provides an opportunity to study irregularities in islet densities. By computer-assisted clustering of islets ≤ 300 µm from each other in 3D space (see Fig. 3b, c and Supplementary Fig. 5), we identified both high islet density regions (HIDR), defined as consisting of >100 islets, and low islet density regions (LIDR) Notably, HIDRs were consistently observed in the periphery of the gland in pancreata from both ND (*n* = 5) and T2D donors (*n* = 2) (Fig. 3a–c, Supplementary Movie 5). Commonly, they were localized in regions in which the acinar tissue appeared disrupted by fibrosis and/or adipocytes, be it in pancreata from ND or diabetic donors. This could be confirmed by complementary LSFM imaging, and by analyses of sections of the investigated samples post-3D-imaging (Fig. 3d–f, Supplementary Movie 6). These observations suggest that peripancreatic inflammation may be a common feature of the human pancreas. In the analyzed pancreas, we observed an islet volume and islet count around three times larger than the average in the HIDRs (Fig. 3g, h, *p* < 0.0001). When analyzing 1945 cm^3^ of ND donor tissue, our data indicate that a substantial portion (around 40%) of the total islet count was contributed by HIDR´s (Supplementary Fig. 5g). Similar patterns were observed in other pancreata, indicating that the islet mass distribution in the human pancreas is heterogenous not only between different regions along its head to the tail axis, but that it also varies along the radial axis with sharp boundaries between HIDRs and LDIRs.

### The autofluorescent properties of the human pancreas can be used as contrast for assessments of 3D anatomy and pathological features

The autofluorescent (AF) properties of tissues can be used as a source of contrast to delineate specific cells or cellular processes^14,15^. In rodent models, internal islet hemorrhaging is associated with T2D and affected islets are suggested to respond less pronounced and more variable to glucose stimulation^16^. Islet hemorrhaging has also been described in the human pancreas where the affected islets negatively influence islet isolation yields for transplantation^17^. Taking advantage of the AF properties of red blood cells (RBCs), we identified hyperemic islets within or in direct conjunction with the antibody labelled regions (Fig. 3i–k, Supplementary Movie 7 and 8). Although the impact of these lesions on human islet physiology remains to be elucidated, the method provides a mean to characterize this phenomenon in larger tissue volumes of the human pancreas and potentially its impact on islet pathophysiology and diabetes disease progression.

As depicted in Fig. 2h, the AF properties of vessels and ducts can be used as a source of contrast to delineate these structures in 3D. Interestingly, by studying the difference in morphology between hyper- and hypointense tubular structures, veins and arteries can be recognized without the need for labelling procedures, arteries having a thicker epithelial wall with strong AF properties. This observation could be confirmed by H&E staining’s post 3D imaging (see Supplementary Fig. 6a and Supplementary Movie 7 and 8). Further, by rendering the tomographic images binary, hypointense regions (i.e. regions with absent or very low signal intensity) could be delineated in 3D by computational processing (see methods for details) (Fig. 3l–o). By algorithm-assisted shape-category selection, various features of the pancreatic anatomy could further be selected for, such as the lumen of tubular structures, based on their elongated shape, and fat deposits based on their spherical appearance (Fig. 3o, Supplementary Fig. 6 and Supplementary Movie 9, see also Fig. 3d, f). Hence, in addition to vessels and ducts, the developed approach can be used to visualize and quantify anatomical features, within maintained organ context throughout the volume of the organ, solely based on their AF properties.

## Discussion

Imaging techniques such as OPT and LSFM have revolutionized our possibilities to study large biomedical tissue specimens in 3D in high resolution. These techniques are continuously evolving, enabling even larger specimens to be studied in high detail. Furthermore, examples of imaging tissue specimens on the human organ scale have been presented based either on the transgenic expression of fluorescent proteins (pig pancreas) or on the investigated tissues auto-fluorescent properties (brain)^18^. Still, for imaging of specifically labelled cell types (in particular by antibodies), these techniques remain limited due to the limited penetration of the labeling agents.

Here, we have established a method to extract the volumes and spatial 3D coordinates of antibody labelled cells within the context of an entire human organ, building on the combination of streamlined procedures for high-throughput tissue-preparation of cm^3^-sized tissue cuboids (using 3D printed matrices for pre-determining tissue size while maintaining spatial context). By staining and imaging of multiple cuboids, followed by stitching of the resultant tomographic data sets back into 3D space, the method circumvents the need to obtain deep tissue labeling throughout the intact organ. Furthermore, it can be performed with the most currently available systems for OPT and LSFM imaging, be it commercial or self-assembled setups^19–21^. Hence, in analogy to a 3D jig saw puzzle, essentially any organ or tissue volume could be encompassed with antibody labelling against selected antigens. In order to cover a complete organ with the size of a pancreas (≈ average volume 73 cm^3^)^22^ or a spleen (≈average volume 215 cm^3^)^23^, a scanning effort similar to the one employed in previous studies evaluating murine diabetes models is required (see e.g^16,24–26^). On the other hand, smaller organs like the thymus and the ovaries (roughly in the range of 5–10 cm^3^ depending on age)^27,28^, would be possible to scan, reconstruct and analyze in about a week after immunolabelling. We propose that, with currently available techniques for optical quantitative 3D assessments of the large tissue specimens, the outlined approach enables the spatially and quantitatively most reliable results to clarify pathological mechanisms in the context of an entire human organ volume. Still allowing for the full freedom of target selectivity that is empowered by the use of antibody labelling of ex vivo preparations.

Generally, LSFM devices provide higher lateral but lower axial resolution as compared to OPT^5^, resulting in non-isotropic voxels, which may result in ambiguities for 3D analysis. Although both LSFM and OPT produced results (on islet dimensions) that were comparable to histological sections when analyzed in 2D (Supplementary Fig. 3), we used OPT as the primary imaging modality in this proof-of-principle study due to its inherent properties of producing isotropic voxels and shorter scan times^29^. As new LSFM setups, approaching isotropic voxels^11^, are being developed, these methods will be well suited for quantitative analysis implementing our approach, with the possibility to provide subcellular resolution. Obviously, any such undertaking will require a careful balance between scan times and data size. As mentioned, numerous clearing and whole-mount immunohistochemistry protocols have been developed for deep-tissue optical imaging (for review see^6–8^). Noteworthy, the whole mount tissue processing and staining methods used in our study are not radically different from previously published protocols for labeling mouse organs^30,31^. Our applied protocol differs mainly in increased incubation times and temperature (see methods) and may therefore be easily implemented in most research environments.

When applied to the pancreas, the described method provides a mean to study the overall impact on islet mass in the pancreas and its spatial distribution in relation to diabetic conditions. Generally, it may elucidate whether islets of different size categories, make-up, or localization exhibit variable pathophysiology in relation to particular disease conditions on an unprecedented scale at the current resolution. The 3D relationship between islets is crucial in orchestrating metabolic adjustment^9^. Previously, OPT-based 3D assessments have demonstrated that the islets in mice are heterogeneously distributed both within (larger islets primarily in the core) and between (differences in islet mass density and numbers) the primary lobular compartments of the pancreas^13,32^. Importantly, our study provides 3D evidence that also the human islet mass may exhibit significant heterogeneities in its distribution, both along the longitudinal and radial axis of the gland, and it confirms stereological indications of the appearance of islet clusters in the organ periphery^9^. In previous studies analyzing slices of the human pancreas, it was not clear if such high islet density regions represent a general feature of the human pancreas. In our study, we identified islet clusters in all investigated pancreata, be it from non-diabetic (*n* = 5) or T2D diabetic (*n* = 2) donors. As demonstrated by LSFM analyses using the AF properties of vessels for contrast, these HIDR´s are highly vascularized (Supplementary Movie 6). In some cases, they were located outside the exocrine parenchyma in fibrotic/adipocyte rich areas (see Fig. 3d, e), supporting the notion^9^ that these HIDR’s may form as a result of continuous but modest inflammation in the periphery of the gland resulting in a local enrichment of islets. It will be of great interest to investigate if these islet clusters have endocrine responses or other functional features that are different from the rest of the islets of the pancreas, and their appearance may have implications for anything from islet transplantation protocols to the development and interpretation of non-invasive islet imaging schemes. Our imaging scheme further provides an abundance of 3D information on islet size and morphology. Notably, we observe a dramatic change in islet sphericity in larger islets in the T2D material (Supplementary Fig. 4). This may be attributed to a rearrangement of endocrine cells within the islets (including an increased proportion of alpha cells), the occurrence of cells lacking cytosolic hormones^33^, or internal islet haemorrhaging^34^, as reported here. These factors, by themselves or together, may contribute to the impaired islet function as previously reported^35^.

Although insulin-specific antibodies were used to generate tissue contrast here, essentially any antibody marker could be used, by itself or in combination with others, to provide a global view on a diverse range of cellular and molecular features including e.g., islet maturity and function. An interesting possibility in this respect is the potential to also quantify expression intensities in the 3D data sets, as recently demonstrated in a mouse model of diabetes^26^. As shown here, OPT and LSFM imaging assessments of specifically labelled targets could be accompanied by a range of structural and pathological features based on the AF properties of the tissue. Recently, we have demonstrated that the hypointense signal of pancreatic ductal adenocarcinoma (PDAC) tissue could be used to delineate tumor borders in 3D^15^. We foresee that the presented approach, with high resolution and target selectivity, may significantly facilitate analyzes of the pancreatic cancer microenvironment. This would considerably affect our possibilities to assess and comprehend the organization of the stromal compartment surrounding the cancer cells, including details on how the cancer-associated fibroblasts, immune cells, nerves, vessels, and lymphatics are organized in a tumor context. Be it for the pancreas or other human organs, the presented method provides an approach to assess its 3D anatomy/pathology with, in theory, unlimited freedom of target selectivity for contrast with currently available technology.

## Methods

### Organ isolation and processing

Pancreata were obtained from diseased donors within the framework for the Nordic Network for Clinical Islet Transplantation (NNCIT). For the samples displayed in Figs. 1–3, the following clinical parameters describing the donors disease history applied with the ND donor listed first and the T2D second; Sex: male, male; Donor age: 69, 66; BMI: 27.5, 34.3; HbA1c: 35, 83; Diabetes duration: NA >10 years; glucose-lowering therapy: None, (Dapagliflozin Metformin, Insulin, Detemir); Donor cause of death: Donation after brain death (DBD), DBD; Warm ischemia time: none, none; Cold ischemia time: 5.5 h, 6 h. The donated pancreata were dissected from the duodenum in Ringer’s acetate (Braun Melsungen AG Hessen Germany) and washed in 1× PBS (Medicago AB, Uppsala, Sweden) before fixation in formaldehyde (Solveco, Rosersberga, Sweden). After 24 h the formaldehyde was replaced with a fresh formaldehyde solution and fixed for another 24 h, followed by stepwise dehydration into ethanol (2 × 75% v/v and 2 × 96% v/v, VWR chemicals) at 4 °C. At this stage, the pancreata were stored and transported in 96% ethanol (v/v) at room temperature. The organs were extensively washed on an orbital shaker in 96% (v/v) ethanol at 4 °C. The ethanol was replaced daily until the alcohol stayed transparent after washing. At this stage, images of the organs (see Supplementary Fig. 1) were obtained with a Nikon D5200 camera.

### Tissue preparation for 3D imaging

All pancreata were scanned in a 64-slice Computed Tomography (CT) scanner (General Electric PET/CT 690) in order to quantify the volume of the organ and generate a reference for downstream analysis. The resolving DICOM files were transformed into.ims files using the Imaris file converter software and uploaded to Imaris (v9.5.1, Bitplane, UK) for iso-surfacing to be used as a 3D spatial template (see Fig. 1). Based on time frames for sufficient antibody penetration, imaging resolution, and organ size, a slicing Matrix was designed with a grid size of 0.55 cm × 0.33 cm (Tinkercad, Autodesk, USA) and 3D printed (Prusa i3 MK3S, Prusa Research, Czech Republic). The pancreata were mounted in 37 °C warm 1.5% low melting point agarose (Lonza™ SeaPlaque™ Agarose, cat: 50100, Lonza, USA) in the 3D printed Matrix. They were subsequently cut into cuboids and the agarose was removed. Tissue cuboids higher than 2 cm were cut in two to facilitate fitting within the NIR-OPT scanner. Images were taken during the slicing process for documentation of X, Y, Z coordinates for each cuboid (see Supplementary Fig. 1). Each biopsy was stored in 100% Methanol (cat: 67-89-4, Fisher Scientific, Sweden) at −20 °C for further processing. 10–15 adjacent tissue cuboids from regions 1–4, (see Fig. 2g, h) were washed thoroughly for 8 h each in 100% methanol on rotation to extract excessive lipids. The samples were brought to −80 °C in 100% MeOH, 3–5 times for at least 1 h for each step, and back to RT to facilitate permeabilization of reagents. In addition, to bleach pigmented cells and reduce autofluorescence, each sample was incubated in a bleach solution containing H_2_O_2_:DMSO:Methanol (3:1:2 respectively)^30^ overnight, followed by a final incubation in freshly made bleach solution for 6–8 h and washed twice in 100% MeOH.

For whole-mount immunolabelling, the samples were rehydrated stepwise from MeOH to TBST (0.15 M NaCl, 0.1 M Tris-HCl pH 7.5, 0.1% Triton^®^ X-100 (cat: 108603, Merckmillipore, Germany), NaCl and Tris-HCl produced in-house (TBK, Norrlands Universitetssjukhuset, Umeå)). Next, the pancreata were incubated in a blocking solution (TBST containing 10% heat-inactivated goat serum (cat: CL1200-500, Cedarlane, Canada) and 5% Dimethyl sulfoxide (cat: D5879, Sigma-Aldrich, Merck KGaA, Germany) and 0,01% sodium azide (in house produced by TBK Norrlands Universitetssjukhus, Umeå)) for 2 days. The same buffer was also used for all antibody incubation steps. After blocking, the samples were incubated with primary antibody (Insulin/Pro-insulin guinea pig (PROGEN Biotechnik GmbH Germany, cat. no. 16049, diluted 1:3000) for 14 days at 37 °C. Following primary antibody incubation, the samples were rigorously washed in 5 × 1 h in TBST on a rotator. Incubation with secondary antibody (anti-guinea pig IRDye 680 Li-Cor, USA, cat: 926-68071, diluted 1:250) was performed as for the primary antibody. The secondary antibody solution was filtered through a 25 mm Acrodisc^®^ w/0.45 μm syringe filter (cat: 4614, Pall Corporation, USA) to remove potential artifacts from fluorophore precipitates. Following antibody labeling, the samples were cleaned from potential artifacts (like tiny fibers and other residues) under a stereomicroscope (Nikon SMZ1500, Japan), washed in distilled water and mounted in 1.5% low melting point agarose (Lonza™ SeaPlaque™ Agarose, cat: 50100, Lonza, USA) at 37 °C, and the agarose was allowed to set for 3–4 h at 4 °C. The tissue cuboids were subsequently dehydrated by 5 cycles of 100% methanol washes on rotation for 1–3 h each. Once fully dehydrated, the specimens were optically cleared using a 1:2 mixture of benzyl alcohol (cat: 100-51-6, Merck, Germany) and benzyl benzoate (BABB) (cat: 105860010, Acros organics, USA), which was replaced 5 times over a time course of 2 days before optical scanning.

### Near infra-red Optical projection tomography (NIR-OPT) and Light sheet fluorescence microscopy (LSFM) imaging

The mounted pancreata were scanned essentially as described^31^ in an in-house build Near-infrared OPT scanner, built on a Leica MZFLIII stereomicroscope (Leica, Wetzlar, Germany) attached to a CoolLED pE-4000 LED Fluorescence Light Source (Ludesco Microscoped, USA) and an Andor iKon-M (Andor technology, Great Britain) camera with a tilted mirror and step motor for sample rotation, while submerged in BABB. A zoom factor of 1.25× were used for all samples, which rendered a pixel size of approximately 21 µm. Filter sets used were Ex: HQ 665/45 nm, Em: HQ 725/50 nm and Ex: 425/60 nm, Em: 480 nm for the stained β-cells and AF channel respectively. Exposure times for individual channels and other scan parameters are listed in Supplementary Table 1. Selected pancreatic cuboids previously scanned by NIR-OPT were reimaged for higher resolution by an UltraMicroscope II (Miltenyi Biotec, Germany) including a 1× Olympus objective (Olympus PLAPO 2XC) coupled to an Olympus MVX10 zoom body, providing 0.63× up to 6.3× magnification with a lens corrected dipping cap MVPLAPO 2× DC DBE objective. In general, a tile scan of 1 × 2 in x and y with 20% overlap at 1× magnification was acquired. In addition, left and right light sheets were “blend” merged with a numerical aperture of 0.14 resolving a thickness of 3.87 µm and 60% width, while using a blending dynamic focus of 10–15 steps across the field of view. Sectional.tif images were generated by the ImspectorPro software and stitched together using the implemented TeraStitcher script (version 9, https://github.com/abria/TeraStitcher) in ImspectorPro (version 7.0.124.0 Lavision Biotec GmbH, Germany). The resulting stitched images were directly converted to.ims files using the Imaris file converter (v9.5.1, Bitplane, UK).

### Post NIR-OPT image processing and 3D reconstruction

For post-NIR-OPT processing and 3D reconstruction, all generated images were processed identically (see Supplementary Table 1 and Supplementary Fig. 2). In order to increase the signal to noise (S: N) ratio of the labelled islets against the surrounding tissue the following steps were performed. First, the pixel range of acquired NIR-OPT frontal projections was adjusted to display minima and maxima. Second, contrast limited adaptive histogram equalization (CLAHE)^32^ algorithm, with a tile size of 32 by 32 for the near-infrared channel (Insulin stain) and a tile size of 16 by 16 for the “anatomy” channel (Ex: 425/60 nm, Em: 480 nm), was implemented to increase the S: N ratio. Additionally, for the insulin channel, a script for image-fusion^36^ of low range, middle range, and high range cut pixel ranges of the same images was applied. The axis of rotation was centered computationally post-scanning using a Discrete Fourier Transform Alignment algorithm (DFTA)^37^. The processed data sets were reconstructed to tomographic sections using the NRecon v1.7.0.4 software (SkyScan Bruker microCT, Belgium) with additional misalignment compensation and ring artifact reduction. The resulting reconstructed.bmp images were converted to.ims files using the imaris (v9.5.1, Bitplane, UK) file converter.

### Spatial and quantitative assessments of islet distribution

Pre-processed OPT images and LSFM images were uploaded to the Imaris Arena (v9.5.1, Bitplane, UK) for spatial and quantitative assessments. Each sample was oriented individually and cropped in 3D. An automated batch processing pipeline was applied (see Supplementary Fig. 2 and Supplementary Table 1). This included an automated baseline subtraction, followed by applying an iso-surfacing algorithm on the “islet” channel (Ex: HQ 665/45 nm, Em: HQ 725/50). The average 3D diameter of a human islet (110 µm) was calculated from the average of 197.097 islets and a region growing seed point filter with this diameter was applied to segment touching islets. A voxel filter, excluding surfaces <5 voxels (approximately corresponding to an object < 46.3 µm^3^). The resulting data was extracted in excel format for statistical analysis and graphs were generated in GraphPad Prism (version 8.4.3, GraphPad Software, LCC).

### Comparative analyses of islet diameters by OPT, LSFM and stereological sectioning

OPT and LSFM sections were matched with corresponding hematoxylin/eosin-stained histological sections obtained post-3D imaging. Islet diameters were calculated by subjectively measuring the x and y diameter for each islet by matching morphological characteristics recognizable for each islet and section plane. The average diameters (*n* = 30) were plotted for each modality (see Supplementary Fig. 3).

### Cluster analysis

For assessments of islet clustering (see Fig. 3b, c and Supplementary Fig. 5), a spot clustering analysis using the Imaris Arena (v9.5.1, Bitplane, UK) batch pipeline editor was performed. The estimated diameter of the average human islet (110 µm, see above) was used for point creation parameters. Hereby, a spot analysis algorithm, including background subtraction and thresholding based on “quality” at above 1 as a statistical value type was implemented. Spot analysis and islet iso-surfacing rendered a non-significant difference in islet count (see Supplementary Fig. 3d–f). The generated spots were split into subgroups with an arbitrary thresholding distance of > 300 µm (based, using the MATLAB (MathWorks, USA)) extension “split spots”. Based on the spot classification, an ROI with a size of 48 voxels (with 21 µm per pixel, corresponding to a cube approximately 1 × 1 × 1 mm) was placed in cluster groups consisting of >100 islets or randomly within the cuboid, and the islet volume and islet number were quantified within these regions. The volume of the ROI was used to normalize islet volume and number (see Fig. 3g, h, respectively).

### Calculation of cuboid volumes and normalization of islet densities

The volumes of the tissue cuboids were calculated in two different ways. In order to compare human islet densities with mouse data, the overall “outline” anatomy volume per cuboid was generated by manually drawing the contour on every 10th slice using the isoline drawing mode and using the autofit function in Imaris to fill in the slices between based on the OPT “anatomy channel” (Ex: 425/60 nm, Em: 480 nm, see Fig. 2d–f). I.e., the calculated volume includes non-tissue-filled regions within the cuboid. Alternatively, when normalizing against tissue volume for human samples (Fig. 2i–k), an anatomy iso-surface was performed based on the “anatomy” channel (Ex: 425/60 nm, Em: 480 nm), using the automated surface algorithm (see above) with a 15 µm “smooth texture” grain size and manual thresholding (see Supplementary Table 1). Hence, hypointense (non-tissue filled) regions were excluded (see Fig. 3l–o).

### Determination of non-labelled tissue volumes based on hypo- and hyperintense tissue fluorescence

#### Hyper intense regions

For iso-surfacing of the volume constituted by internal islet hemorrhaging, thus the “anatomy” channel (Ex: 425/60 nm, Em: 480 nm) was masked onto the surfaced islets (see spatial and quantitative assessments of islet distribution above). Hereby, the AF signal originating from RBC’s within islets was segmented (Fig. 3i) as for the antibody labeled islets (see above). AF originating from vessels and ducts was iso-surfaced similarly (see Supplementary Fig. 6).

#### Hypointense regions

Hypointense regions (i.e., regions with low AF properties or lacking tissue), enclosed by hyperintense regions were segmented by applying a binary filter to the tomographic sections, and inverting the pixel range using the Imaris image processing function (v9.5.1, Bitplane, UK) (Fig. 3 l–n). By masking the inverted binary image onto the outline anatomy (see above) the hypointense areas could be visualized in 3D. For the classification of spherical and tubular characteristics (fat deposits and the lumen of vessels and ducts), we implemented the sphericity filter in Imaris (see Fig. 3o and Supplementary Fig. 6).

### Stitching of individual data sets in 3D space

The 3D volumes of the non-overlapping cuboids were stitched together in Imaris using post-slicing reference images (Supplementary Fig. 1). Hereby, each data set was added individually to the workspace and the AF from the vascular and ductal system was used to facilitate alignment (Fig. 2h).

### Post 3D imaging histology

For validation of islet clustering and intra-islet hemorrhaging, the tissue cuboids were dehydrated to 96% ethanol (cat: 20823.362, VWR International, USA) post 3D imaging, paraffin-embedded, sectioned, and stained for hematoxylin/eosin staining using an automated Ventana Benchmark staining machine (Ventana Medical Systems, Tucson, AZ, USA). The stained sections were compared to optical sections from OPT and LSFM imaging (Fig. 3 and Supplementary Figs. 3 and 6).

### Statistics and reproducibility

All statistical data from individual samples (segmented islets, segmented anatomy, and islet clustering were exported from Imaris as to Excel® (Microsoft^®^, office 365, version 2008), using the query import metadata function for multiple files. For sorting purposes, each islet received an individual ID, sample ID, and Pancreas ID. By averaging the islet´s axis length in x, y, and z of each individual islet, the mean islet 3D diameter was calculated (Fig. 2a–c). Stereological data (distribution of islet volumes and numbers) was obtained from Hellman 1961^12^ and data on mouse islet distribution in healthy C57Bl/6 at 8 weeks was obtained from Parween et al., 2017^38^. Islets were sorted into size categories (average 3D diameter, Fig. 2b, c) or volume (Fig. 2e, f, and j, k), using the below in-house written python script in Jupyter Notebook (Project Jupyter, USA). Annotations of individual lines are indicated with the ‘#‘ operator. Pandas and Numpy libraries are required to run the code.

import pandas as pd

import numpy as np

#edit here your input path and file name. An excel file with individual islet parameters was used here as input.

data = pd.read_excel (r’C:/Users/Max_Hahn/Projects/Human_pancreas/3D_islet_quantification/islet_paramters_T2D_all_regions.xlsx’)

df = pd.DataFrame(data)

#choose islet size category intervals (in volume [µm^3]) here (as shown in graphs in Fig. 2 e, f, j and k).

df[‘islet size categories’] = pd.cut(df[‘Islet Volume [µm3]’], [0,25000,50000,100000,200000,400000,800000,1600000,3200000,6400000,12800000,25600000,51200000,102400000])

#For graphs in Fig. 2 b, c, the below intervals for mean 3D islet diameter [in µm] were used.

#df[‘islet size categories’] = pd.cut(df[‘mean_diameter’], [0,62.5,78.1,93.7,109.3,140.6,171.9,203.2,234.5,265.8,297.1,328.4])

#sort here individual islet size categories for pancreas region and sample ID. The islet volume sum of each interval is then calculated.

df_stacked = df.groupby([‘Region ID’, ‘islet size categories’, ‘Sample ID’]).agg({‘Islet Volume [µm3]’: np.sum, ‘Sample ID’: ‘count’}).transpose().stack().fillna(0)

df_stacked.rename(index = {‘Islet Volume [µm3]’: ‘sum [µm^3]’, ‘Sample ID’: ‘count’}, inplace = True)

#print(df_stacked)

#edit here your output path and name your output excel file.

df_stacked.to_excel (r’C:/Users/Max_Hahn/Projects/Human_pancreas/3D_islet_quantification/islet_paramters_T2D_all_regions_py_sorted.xlsx’, index = True, header = True).

Graphs were generated, and significance tests were performed in GraphPad Prism (version 8.4.3, GraphPad Software, LCC). For Fig. 2d and Fig. 3g, h an ordinary one-way ANOVA significance test with alpha level set to 0.05 and Tukey’s multiple comparison post-hoc test was used. For Fig. 2i a non-parametric Kruskal–Wallis test with Dunn´s post-hoc multiple comparisons test was used. A non-parametric Wilcoxon matched pair test was used for islet cluster comparison (Supplementary Fig. 4g) and a two-way ANOVA with Šídák’s multiple comparisons test was used to compare the mean sphericity between islet size subgroups of the ND and T2D samples (Supplementary Fig. 4).

### Ethics declaration

All work involving human tissue was conducted according to the principles expressed in the Declaration of Helsinki and in the European Council’s Convention on Human Rights and Biomedicine. Consent for organ donation for use in research was obtained from the donor prior to death via the Swedish National Donor Registry (https://www.socialstyrelsen.se/en/apply-and-register/join-the-swedish-national-donor-register/) or from the relatives of the deceased donors by the donor’s physicians and documented in the medical records of the deceased subject. The study was approved by the Regional Ethics Committee in Uppsala, Sweden.

### Reporting summary

Further information on research design is available in the Nature Research Reporting Summary linked to this article.

## Supplementary information

Supplementary information

Description of Supplementary Files

Supplementary Movie 1

Supplementary Movie 2

Supplementary Movie 3

Supplementary Movie 4

Supplementary Movie 5

Supplementary Movie 6

Supplementary Movie 7

Supplementary Movie 8

Supplementary Movie 9

Supplementary Data 1

Reporting Summary

## Data Availability

Source data for the graphs and charts in the main figures are available as Supplementary Data 1. Any remaining information, including Raw and processed imaging datasets acquired by NIR-OPT and LSFM on all samples displayed are available from the authors on reasonable request.

## Code availability

OPT acquisition and analysis code^32,36,37^ are available from the authors upon request, subject to an MTA.
