# Supplementary material for: 3D imaging of human organs with micrometer resolution - applied to the endocrine pancreas
Source: Commun Biol. 2021 Sep 10;4:1063. doi: 10.1038/s42003-021-02589-x (PMC8433206; doi:10.1038/s42003-021-02589-x)
Supplement: Supplementary file 1 — Supplementary information [file 42003_2021_2589_MOESM1_ESM.pdf]

Supplemental information for

3D imaging of human organs with micrometer resolution - applied to the endocrine pancreas

Max Hahn[1], Christoffer Nord[1], Maria Eriksson[1], Federico Morini[1], Tomas Alanentalo[1], Olle Korsgren[2] & Ulf Ahlgren[1,*].

[1]Umeå Centre for Molecular Medicine, Umeå University, Umeå, Sweden.
[2] Dept. of Immunology, Genetics and Pathology, Uppsala University, Uppsala, Sweden.

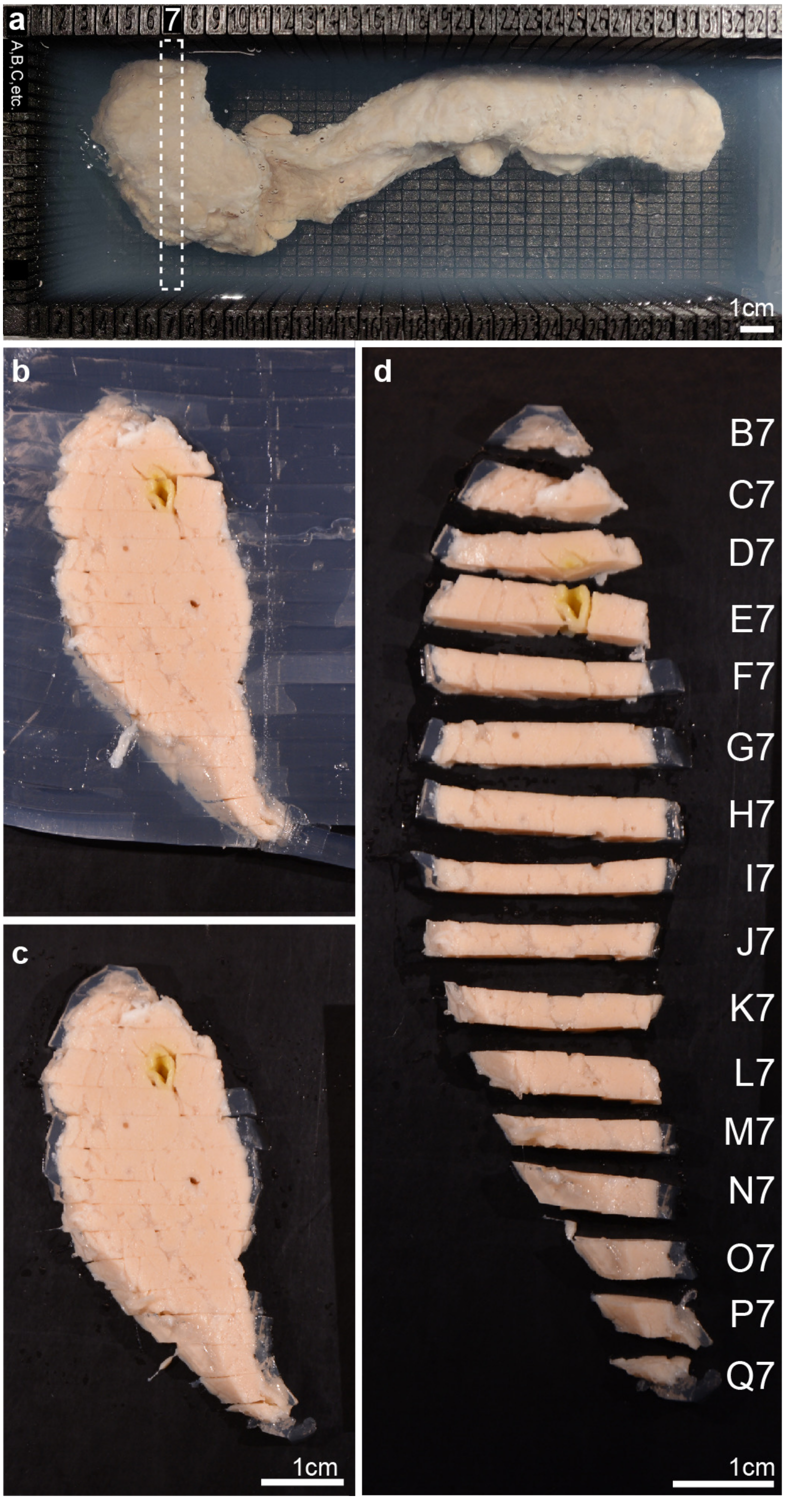

Supplementary Fig. 1. a-d, Image series illustrating the subdivision of an intact pancreas into tissue cuboids for 3D immunohistochemistry, retaining information of their spatial origin within the organ. By this technique an organ on the current scale may be specifically antibody labelled and 3D imaged by OPT or LSFM. The organ is allowed to set in a 3D printed (a)matrix (grid size in this example 0.55. x 0.35 cm in x-y) and cut by a pathological blade into cuboids. The cut cuboids (b) are cleaned from agarose (c) and processed individually (d) for whole mount immunohistochemistry and NIR-OPT and LSFM scanning.

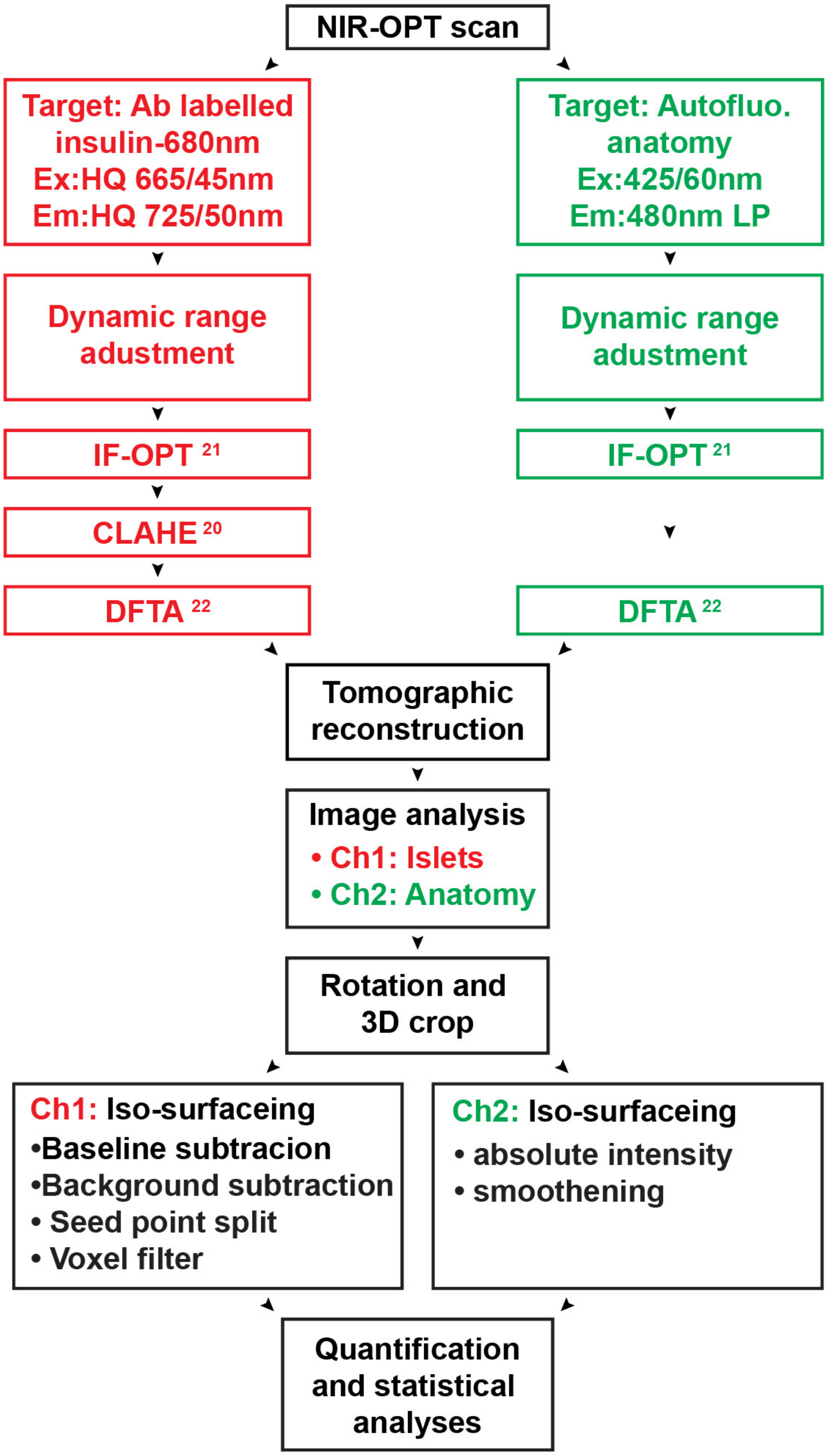

Supplementary Fig. 2. Schematic illustration of the NIR-OPT image processing pipeline for insulin labelled islets (red) and autofluorescent features (green) respectively. See methods for details.

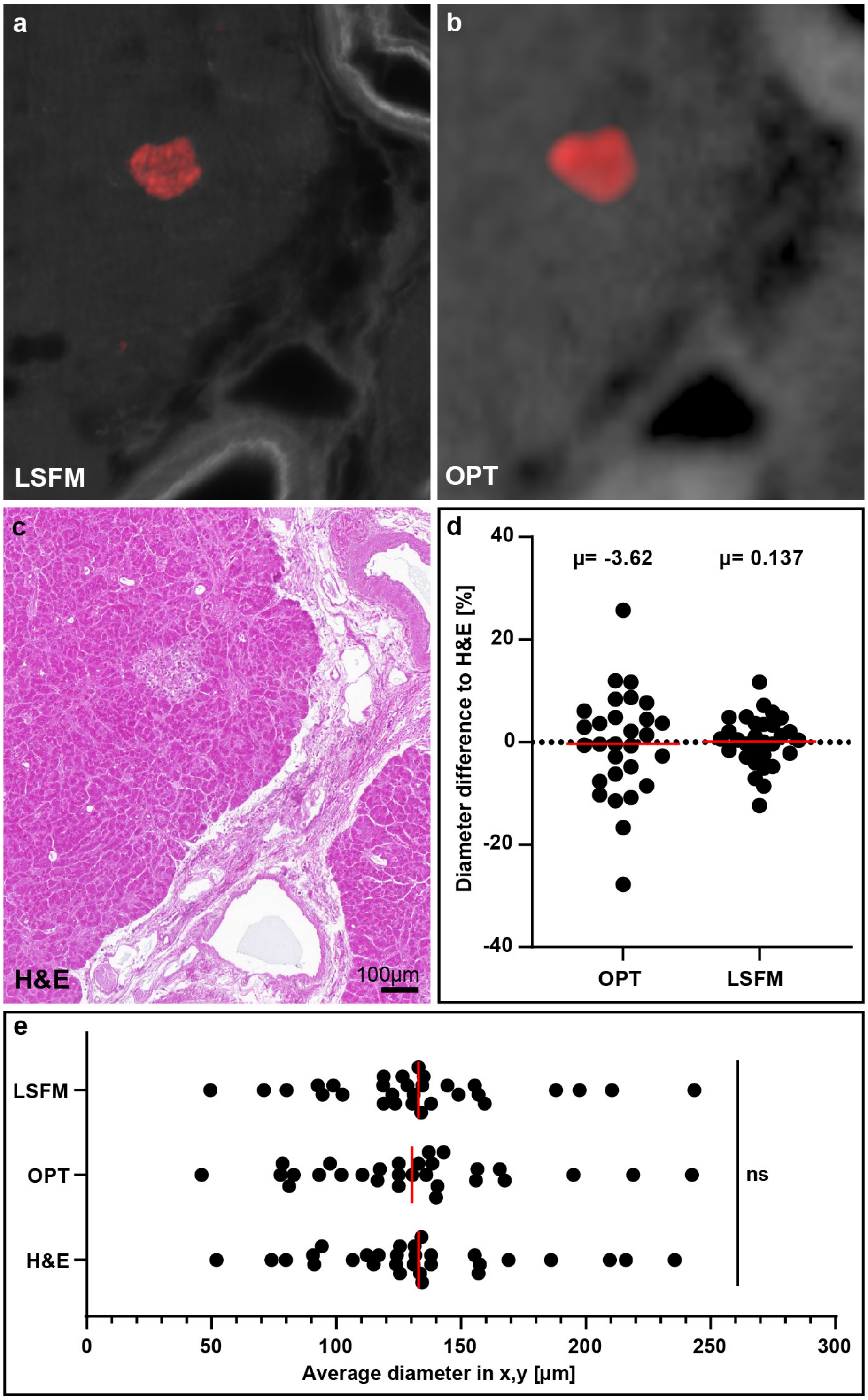

Supplementary Fig. 3. Comparison of islet diameters measured by OPT and LSFM vs stereology. **a-c**, OPT section (a) and corresponding LSFM section (b) showing and islet stained for insulin (red) and a tissue section obtained post 3D imaging stained with hematoxylin and eosin (H&E) of the same area. **d**, Plot showing difference in islet diameters (measured on 2D section planes) between OPT and LSFM compared to diameters from H&E-stained sections (n=30 islets). The average difference (µm) is less than 5 % for both modalities. **e**, Plot showing the average 2D diameter for each measured islet for each imaging technique (LSFM vs H&E, p=0.994, OPT vs H&E, p=0.805, LSFM vs OPT, p= 0.809).

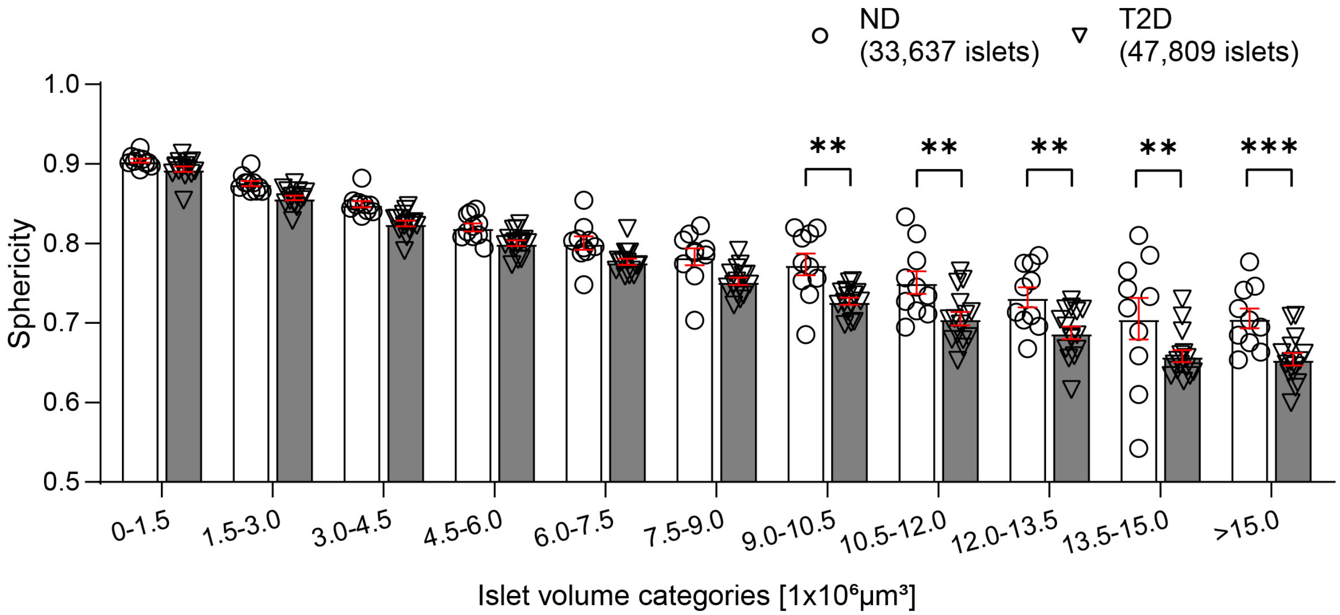

Supplementary Fig. 4. Analysis of human islet sphericity. Graph showing average islet sphericity per size category in 33. 637 islets from a ND donor and 47.809 islets from a T2D donor. A sphericity value of 1 corresponds to a perfect sphere. Each data point corresponds to the average from a tissue cuboid. Generally, for both groups the islet sphericity decreases with increasing islet size, and larger islets are significantly less spherical in the T2D specimen than in the ND specimen ($p<0.01$). Error bars are displayed as ±SEM. Significance was tested using a 2way ANOVA with Šidák's multiple comparison test post-hoc.

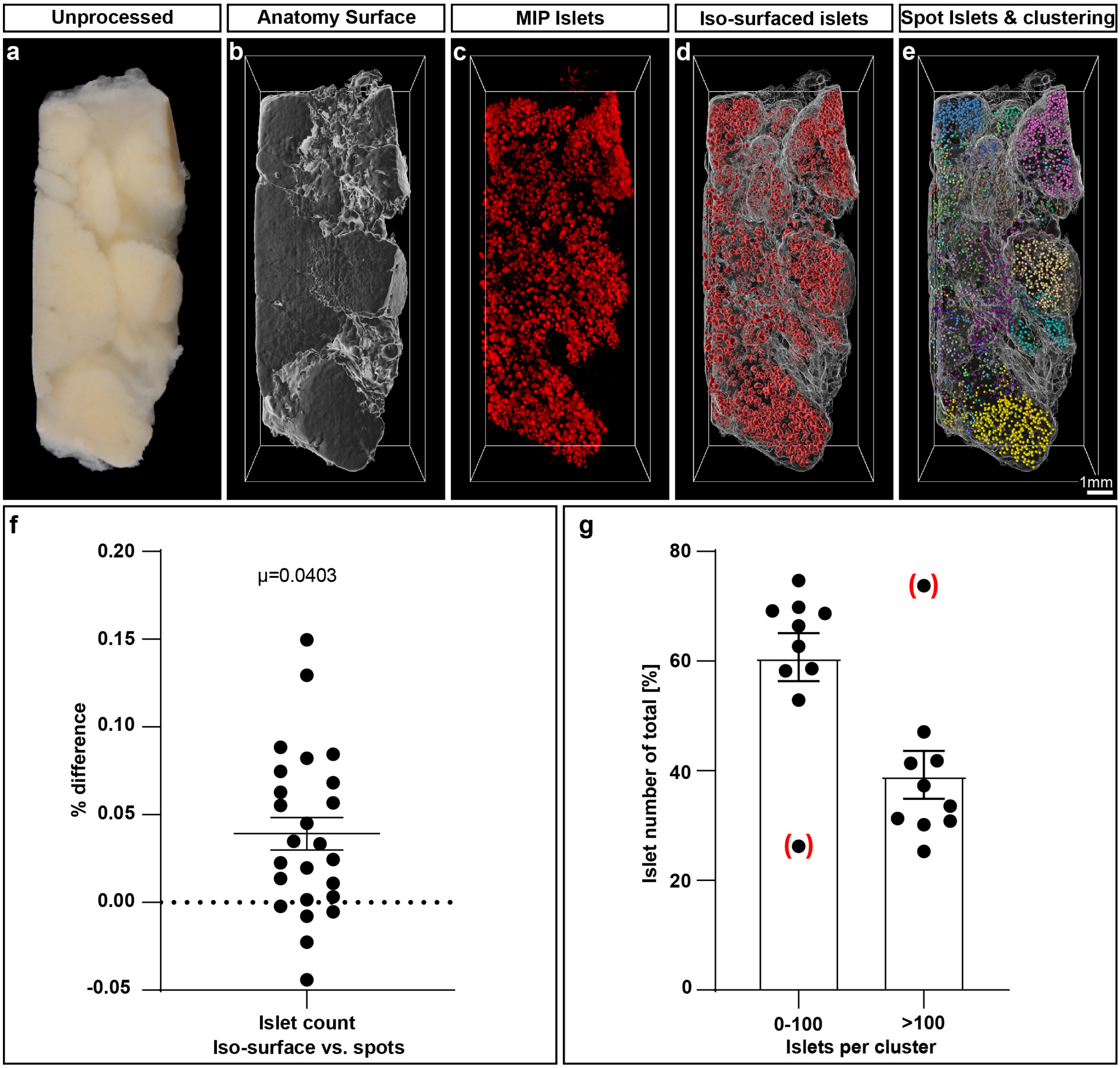

Supplementary Fig. 5. Validation of cluster analysis. **a**-**e**, Images illustrating the cluster analysis workflow, showing an unprocessed tissue cuboid (a), its "anatomical" outline based on tissue AF (b, grey) by OPT, maximum intensity projection (MIP) view of the insulin labelled islets (c, red), the iso-surfaced islets (d, red) and the transparent anatomy (d, grey) and spot assignment and distance clustering (<300μm) (e). **f**, Plot distribution showing the mean difference of the islet count of the surface analysis vs. the spot analysis. Each data point corresponds to all islets of a tissue cuboid encompassing in total 81.446 islets. The mean difference in islet count is ≤ 0.04 % between the analysis methods. **g**, Graph showing islet number distribution between clusters with 0-100 and > 100 islets respectively normalized to the total islet count. Clusters consisting of > 100 islets correspond to approximately 40 % of the total islet count (p=0.0645). Note, when removing the outlier (red brackets in (g)), which corresponds to a small cuboid derived from the very edge of the pancreas (consisting essentially only of a HIDR region), p=0.0039. Significance was tested using a non-parametric Wilcoxon *matched pair test* See methods for details. Error bars represent ±SEM.

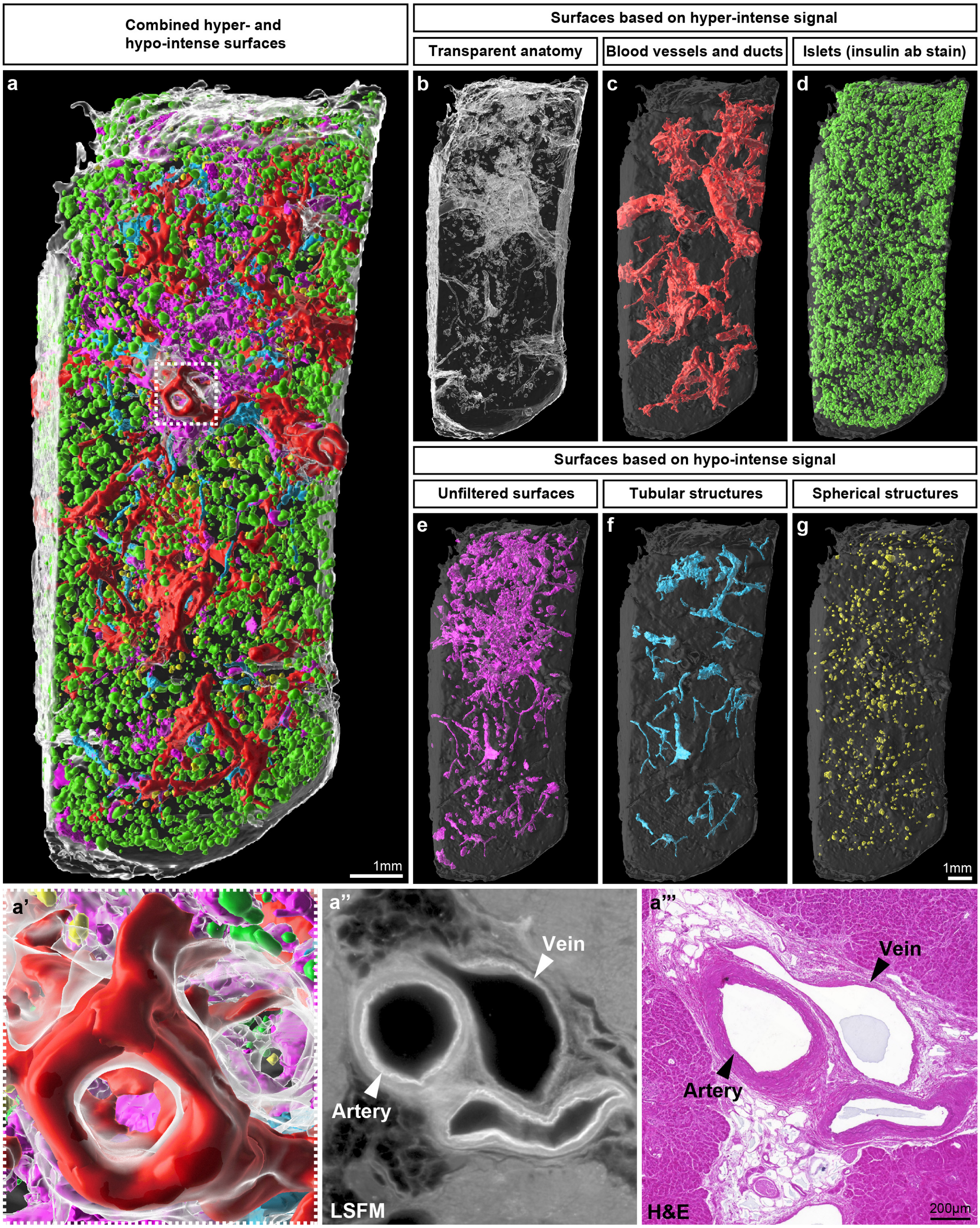

Supplementary Fig. 6. Reconstruction of hypo- and hyper-intense regions. **a**, OPT generated image combining iso-surfaced features seen in (b-g). **b**, transparent anatomy based on hypo-intense regions. **c**, blood vessels and ducts (red) iso-surfaced based on their hyper-intense AF signal. **d**, Islets of Langerhans (green) reconstructed based on insulin antibody staining. **e**, unfiltered hypo-intense surfaces (lilac). **f**, **g**, Sphericity filtered structures based on (e), showing the lumen of tubular structures (f, blue) and spherical hypointense enclosures (g, yellow). **a´,** High magnification of region corresponding to box in (a), sowing an artery (red) and a vein (grey-white) based on their hyperintense and hypo-intense AF properties respectively. **a´´**, 4µm LSFM section corresponding to the region in (a´). **a´´´**, H&E-stained section of the region corresponding to (a´´) obtained post 3D imaging. Arteries and veins can be identified in 3D based solely on the thickness and AF properties of their vascular walls.

Image acquisition and batch pipeline processing			
	Channel	Insulin labelled Ab	Autofluor. Anatomy
Near-infra Red Optical projection tomography	Number of scanned cuboids (human ND & T2D)	55	55
	OPT magnification	1.25 x	1.25 x
	Roation Step (deg)	0,9	0,9
	Image number	400	400
	Voxel size (in xyz)	21.05 µm	21.05 µm
	Exp. time of filter (Ex: 425/60 & Em: 480LP) (target: autofluor. Anatomy)	N.A.	500 -700 ms
	Exp. time of filter (Ex: HQ 665/45 & Em: HQ 725/50) (target:Ab labelled Insulin-680nm)	3000 - 4500 ms	N.A.
Imaris Image analysis	Format Version	9,3	9,3
	Surfaces Creation Parameters		
	[Algorithm]		
	Enable Region of Interest	FALSE	FALSE
	Enable Region Growin	TRUE	FALSE
	Enable Tracking	FALSE	FALSE
	Enable shortest Distance	FALSE	FALSE
	[Source channel]		
	Source Channel Index	1 (Insulin-680)	2 (AF Anatomy)
	Enable Smooth	FALSE	TRUE
	Surface Grain Size	42,0874	15 µm
	Enable Eliminate Background	TRUE	FALSE
	Diameter of largest Sphere	157,828	157,828
	[Threshold]		
	Enable Automatic Threshold	FALSE	FALSE
	Manual Threshold Value	5	15 - 30
	Active Threshold	TRUE	TRUE
	Enable automatic Threshold B	TRUE	TRUE
	Manual Threshold Value B	170,979	235,82
	Active Threshold B	FALSE	FALSE
	Region Growing Estimated Diameter	110 µm	N.A.
	Region Growing Background Subtraction	TRUE	N.A.
	[Filter Seed Points]		
	"Quality" above	1	N.A.
	[Filter Surfaces]		
	"Number of Voxels" filter above	5	10
	Spot Creation Parameters		
	[Algorithm]		
	Enable Region of Interest	FALSE	N.A.
	Enable Region Growin	FALSE	N.A.
	Enable Tracking	FALSE	N.A.
	Enable Region Growing	FALSE	N.A.
	Enable shortest Distance	TRUE	N.A.
	[Source channel]		
	Source Channel Index	1 (Insulin-680)	N.A.
	Estimated Diameter	110 µm	N.A.
	Background Subtraction	TRUE	N.A.
	[Filter Spots]		
	"Quality" above	1	N.A.
	[Split Spots XTension]		
	Spots in sub groups that have Distance > threshold Threshold:	300 µm	N.A.

Supplementary table 1. Table listing image acquisition and post processing parameters.
