# Supplementary material for: 3D imaging of human organs with micrometer resolution - applied to the endocrine pancreas
Source: Commun Biol. 2021 Sep 10;4:1063. doi: 10.1038/s42003-021-02589-x (PMC8433206; doi:10.1038/s42003-021-02589-x)
Supplement: Supplementary file 2 — Description of Supplementary Files [file 42003_2021_2589_MOESM2_ESM.pdf]

Description of Additional Supplementary Files

File name: Supplementary Movie 1
Description: Video of a representative pancreatic cuboid from a non-diabetic (ND) human donor imaged by optical projection tomography (OPT). Islet β-cells labelled for insulin (red) are depicted as 3D Maximum intensity projection (MIP) and as x/z and y/z tomographic sections. The anatomical volume of the cuboid based on the autofluorescence (AF) is displayed in grey.

File name: Supplementary Movie 2
Description: Video of a representative pancreatic cuboid from a non-diabetic (ND) human donor imaged by light sheet fluorescence microscopy (LSFM) as seen in Supplementary Video 1. Islet β-cells labelled for insulin (red) are depicted as 3D Maximum intensity projection (MIP) and as x/z and y/z tomographic sections. The anatomical volume of the cuboid based on the autofluorescence (AF) is displayed in grey.

File name: Supplementary Movie 3
Description: Video of a representative pancreatic cuboid from a type 2 diabetic (T2D) human donor imaged by optical projection tomography (OPT). (Left), 3D Maximum intensity projection (MIP) visualizes islet β-cell labelled for insulin (red). The anatomical volume of the cuboid based on the autofluorescence (AF) is displayed in grey. (Right), the same cuboid showing statistically color-coded islets to delineate islet diameter distribution within a "transparent" rendered AF based anatomy surface.

File name: Supplementary Movie 4
Description: Video of 10 pancreatic cuboids from a type 2 diabetic (T2D) human donor imaged by optical projection tomography (OPT). The 3D Maximum intensity projections (MIP) of the specimens display islet β-cells labelled for insulin (red) and anatomical volumes (grey) and were virtually stitched together post-imaging of individual cuboid scans.

File name: Supplementary Movie 5
Description: Video of 3 adjacent pancreatic cuboids from the tail region of a non-diabetic (ND) human pancreas imaged by optical projection tomography (OPT). Islet β-cell distributions labelled for insulin (red) and anatomical volumes (grey) are displayed as 3D Maximum intensity projections (MIP). To delineate high islet density regions located in the organs surface, islet-based spot clusters were generated and individual clusters were differently color coded (>100 islets per cluster shown only). Within in each cluster, islets are located within 300 µm from its nearest islet neighbor in 3D space.

File name: Supplementary Movie 6
Description: Light sheet fluorescence microscopy (LSFM) video displaying a representative high islet density region (HIDR, islet β-cells labelled for insulin (red)) located in the periphery of the gland as a 3D Maximum intensity projection (MIP) and as x/z and y/z sections. The anatomical volume of the specimen, vessels, ducts, and fibro-fatty replacements based on the autofluorescence (AF) are displayed in grey.

File name: Supplementary Movie 7
Description: Video of a representative pancreatic cuboid harboring intra islet lesions from a type 2 diabetic (T2D) human donor imaged by light sheet fluorescence microscopy (LSFM). Islet β-cells labelled for insulin (red) are depicted as 3D Maximum intensity projection (MIP). Anatomical volume and intra islet hemorrhages are visualized as 3D MIP and as traversing sections based on the autofluorescent (AF) properties of red blood cells and tissue (grey).

File name: Supplementary Movie 8
Description: Video of a representative ROI pancreatic cuboid harboring intra islet lesions from a type 2 diabetic (T2D) human donor imaged by light sheet fluorescence microscopy (LSFM). Islet β-cells labelled for insulin (red) are depicted first as 3D Maximum intensity projection (MIP), followed by orthogonal slices and then as 3D iso-surfaces.

File name: Supplementary Movie 9
Description: Video of a representative pancreatic cuboid from a type 2 diabetic (T2D) human donor imaged by optical projection tomography displaying surfaced hyper- and hypo intense regions as depicted in supplementary figure 6.

File name: Supplementary Data 1.
Description: Source data for individual graphs.
