# Supplementary material for: 3D imaging of human organs with micrometer resolution - applied to the endocrine pancreas
Source: Commun Biol. 2021 Sep 10;4:1063. doi: 10.1038/s42003-021-02589-x (PMC8433206; doi:10.1038/s42003-021-02589-x)
Supplement: Supplementary file 13 — Reporting Summary [file 42003_2021_2589_MOESM13_ESM.pdf]

nature research

Corresponding author(s): Ulf Ahlgren

Last updated by author(s): Jul 23, 2021

Reporting Summary

Nature Research wishes to improve the reproducibility of the work that we publish. This form provides structure for consistency and transparency in reporting. For further information on Nature Research policies, see our Editorial Policies and the Editorial Policy Checklist.

Statistics

For all statistical analyses, confirm that the following items are present in the figure legend, table legend, main text, or Methods section.

n/a	Confirmed	
☐	☒	The exact sample size (n) for each experimental group/condition, given as a discrete number and unit of measurement
☐	☒	A statement on whether measurements were taken from distinct samples or whether the same sample was measured repeatedly
☐	☒	The statistical test(s) used AND whether they are one- or two-sided<br>*Only common tests should be described solely by name; describe more complex techniques in the Methods section.*
☒	☐	A description of all covariates tested
☐	☒	A description of any assumptions or corrections, such as tests of normality and adjustment for multiple comparisons
☐	☒	A full description of the statistical parameters including central tendency (e.g. means) or other basic estimates (e.g. regression coefficient) AND variation (e.g. standard deviation) or associated estimates of uncertainty (e.g. confidence intervals)
☐	☒	For null hypothesis testing, the test statistic (e.g. F, t, r) with confidence intervals, effect sizes, degrees of freedom and P value noted<br>*Give P values as exact values whenever suitable.*
☒	☐	For Bayesian analysis, information on the choice of priors and Markov chain Monte Carlo settings
☒	☐	For hierarchical and complex designs, identification of the appropriate level for tests and full reporting of outcomes
☒	☐	Estimates of effect sizes (e.g. Cohen's d, Pearson's r), indicating how they were calculated

Our web collection on statistics for biologists contains articles on many of the points above.

Software and code

Policy information about availability of computer code

Data collection	In-house developed NIR-OPT software (Eriksson et al., JoVE, 2013) LabVIEW version 20.0f1 (National Instruments, USA), ImspectorPro, version 7.0.124 (LaVision Biotec GmbH, Germany)
Data analysis	DataViewer, version 1.5.6.2 (SkyScan, Bruker microCT); CLAHE (Hörnblad et al., Islets 2011); A-value tuning (Cheddad et al., IEEE Trans. Med. Imaging, 2012); NRecon, version 1.7.0.4 (SkyScan, Bruker microCT); Imaris File Converter x64, version 9.5.1 (Bitplane AG); Imaris x64, version 9.5.1 (Bitplane, UK); Terastitcher v9 implemented, version 7.0.124 (LaVision Biotec GmbH); 3DHISTECH's Slide Converter and CaseViewer, version 2.3 (3DHISTECH, Hungary); for islet classification in Python: Jupyter Notebook (Project Jupyter, USA).

For manuscripts utilizing custom algorithms or software that are central to the research but not yet described in published literature, software must be made available to editors and reviewers. We strongly encourage code deposition in a community repository (e.g. GitHub). See the Nature Research guidelines for submitting code & software for further information.

Data

Policy information about availability of data

All manuscripts must include a data availability statement. This statement should provide the following information, where applicable:

- Accession codes, unique identifiers, or web links for publicly available datasets
- A list of figures that have associated raw data
- A description of any restrictions on data availability

Raw and processed imaging datasets acquired by NIR-OPT and LSFM and all samples displayed are available from the authors upon reasonable request.

Field-specific reporting

Please select the one below that is the best fit for your research. If you are not sure, read the appropriate sections before making your selection.

[x] Life sciences [ ] Behavioural & social sciences [ ] Ecological, evolutionary & environmental sciences

For a reference copy of the document with all sections, see nature.com/documents/nr-reporting-summary-flat.pdf

Life sciences study design

All studies must disclose on these points even when the disclosure is negative.

Sample size	Sample sizes for this methodological proof of principle study is given in the manuscript.
Data exclusions	N/A
Replication	All experimental procedures and findings were successfully replicated on other pancreatic tissues and confirmed by histological/fluorescent antibody labeling experiments
Randomization	No Randomization was performed for this methodological proof of principle study
Blinding	No Blinding was performed or this methodological proof of principle study

Reporting for specific materials, systems and methods

We require information from authors about some types of materials, experimental systems and methods used in many studies. Here, indicate whether each material, system or method listed is relevant to your study. If you are not sure if a list item applies to your research, read the appropriate section before selecting a response.

Materials & experimental systems

n/a	Involved in the study	
[ ]	[x]	Antibodies
[x]	[ ]	Eukaryotic cell lines
[x]	[ ]	Palaeontology and archaeology
[x]	[ ]	Animals and other organisms
[x]	[ ]	Human research participants
[x]	[ ]	Clinical data
[x]	[ ]	Dual use research of concern

Methods

n/a	Involved in the study	
[x]	[ ]	ChIP-seq
[x]	[ ]	Flow cytometry
[x]	[ ]	MRI-based neuroimaging

Antibodies

Antibodies used	Primary:<br>- anti-Insulin/Pro-insulin guinea pig (PROGEN Biotechnik GmbH Germany, cat. no. 16049, diluted 1:3000)<br>Secondary:<br>-anti-guinea pig IRDye 680 Li-Cor, USA, cat: 926-68071, diluted 1:250
Validation	Both primary and secondary antibodies were titrated and validated on positive control sections before application.
